# Combination of Primary Hemostatic Disorders and Atrial Fibrillation Increases Bleeding Events Following Transcatheter Aortic Valve Replacement

**DOI:** 10.1055/a-2068-5783

**Published:** 2023-05-11

**Authors:** Kensuke Matsushita, Benjamin Marchandot, Marion Kibler, Adrien Carmona, Truong Dinh Phi, Joe Heger, Antonin Trimaille, Sébastien Hess, Laurent Sattler, Mickael Ohana, Antje Reydel, Laurence Jesel, Patrick Ohlmann, Olivier Morel

**Affiliations:** 1 Université de Strasbourg, Pôle d'Activité Médico-Chirurgicale Cardio-Vasculaire, Nouvel Hôpital Civil, Centre Hospitalier Universitaire, Strasbourg, France; 2 UMR1260 INSERM, Nanomédecine Régénérative, Université de Strasbourg, Strasbourg, France; 3 Department of Haemostasis, Centre Hospitalier Universitaire, Strasbourg, France; 4 Department of Radiology, Nouvel Hôpital Civil, Centre Hospitalier Universitaire, Strasbourg, France

**Keywords:** transcatheter aortic valve replacement, atrial fibrillation, closure time of adenosine diphosphate, primary hemostasis, bleeding events

## Abstract

**Background** Patients with atrial fibrillation (AF) are likely to have a poor prognosis including bleedings following transcatheter aortic valve replacement (TAVR). Closure time of adenosine diphosphate (CT-ADP) is a primary hemostasis point-of-care test and is a predictor of bleeding events following TAVR. We aimed to evaluate the impact of ongoing primary hemostatic disorders on bleeding events in TAVR patients with AF.

**Methods** We enrolled 878 patients from our prospective registry. The primary endpoint was VARC-2 major/life-threatening bleeding complications (MLBCs) at 1 year after TAVR and secondary endpoint was major adverse cardiac and cerebrovascular events (MACCEs) at 1 year, defined as a composite of all-cause death, myocardial infarction, stroke, and heart failure hospitalization. Ongoing primary hemostatic disorder was defined by a postprocedural CT-ADP >180 seconds.

**Results** Patients with AF had a higher incidence of MLBCs (20 vs. 12%, *p* = 0.002), MACCE (29 vs. 20%, *p* = 0.002), and all-cause mortality (15 vs. 8%, *p* = 0.002) within 1 year compared to non-AF patients. When the cohort was split into four subgroups according to AF and CT-ADP >180 seconds, patients with AF and CT-ADP >180 seconds had the highest risk of MLBCs and MACCE. Multivariate Cox regression analysis confirmed that the patients with AF and CT-ADP >180 seconds had 3.9-fold higher risk of MLBCs, whereas those patients were no longer associated with MACCE after the adjustment.

**Conclusion** In TAVR patients, AF with postprocedural CT-ADP >180 seconds was strongly associated with MLBCs following TAVR. Our study suggests that persistent primary hemostatic disorders contribute to a higher risk of bleeding events particularly in AF patients.

## Introduction

Transcatheter aortic valve replacement (TAVR) has emerged as an established treatment in patients with symptomatic severe aortic stenosis (AS). 1 2 3 4 Atrial fibrillation (AF) is common in patients undergoing TAVR and constitutes an indication for long-term oral anticoagulants (OACs) with a vitamin K antagonist (VKA) or direct-acting OAC (DOAC). The reported prevalence of pre-existing AF in patients undergoing TAVR ranges from 16 to 51%. 5 6 While AF is a strong predictor of thromboembolic events, the benefits of anticoagulation therapy require careful balancing against the increased risk of bleeding events. In fact, numerous studies have stressed that AF was associated with an increased rate of all-cause mortality and bleeding events in patients undergoing TAVR compared with those in sinus rhythm. 6 Notably, a recent observational study conducted by Lother et al demonstrated that the incidence of bleedings defined the in-hospital outcome of patients with AF after transfemoral TAVR. 5 Although studies have suggested that the postprocedural phase demands particular care in bleeding prevention, an indicator of future bleeding events in AF patients is lacking.

Closure time of adenosine diphosphate (CT-ADP) is a point-of-care test used as a surrogate marker of primary hemostatic disorder. We previously established that postprocedural CT-ADP >180 seconds was an independent predictor of significant paravalvular leak (PVL), early- and late-bleeding events, and increased 1-year mortality among patients undergoing TAVR. 7 8 9 Interestingly, our observational study highlighted that CT-ADP >180 seconds was also associated with early ischemic neurological events as well as major bleeding events. 10 Consistent with prior studies, the paradoxical relationship between bleeding and ischemic stroke events can be explained by the low hemoglobin (Hb) level which may induce reduced cerebral oxygenation or blood transfusions contributed to the increased risk of stroke through a transfusion-enhanced systemic inflammatory reaction, causing a prothrombotic environment. 11 12 In line with this paradigm, strategies aimed at reducing the risk of bleeding constitute an important area of patient's care improvement.

Thus, the current study aimed to evaluate the impact of ongoing primary hemostatic disorders, defined by a CT-ADP >180 seconds after TAVR, on bleeding events in TAVR patients with AF.

## Methods

### Study Design and Patients

**Fig. 1.**
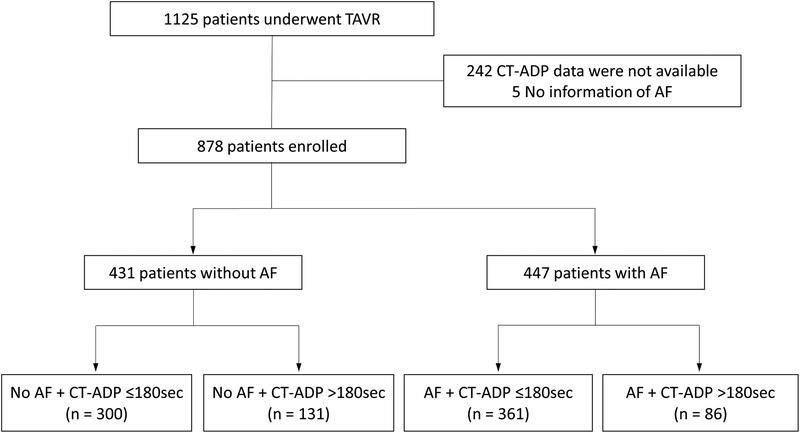
Study design. AF, atrial fibrillation; CT-ADP, closure time adenosine diphosphate; TAVR, transcatheter aortic valve replacement.

Consecutive patients with symptomatic severe AS 13 who underwent TAVR were enrolled in a prospective registry at our institution (Nouvel Hôpital Civil, Université de Strasbourg, Strasbourg, France) between February 2012 and May 2019. For the purpose of the present analysis, absence of CT-ADP assays and patients with missing AF data were excluded ( Fig. 1 ). Indications for TAVR and procedural approaches were assessed by the local heart team. The balloon-expandable Edwards SAPIEN XT or S3 prosthesis (Edwards Lifesciences, Irvine, California, United States), the self-expandable CoreValve, Evolut-R, or Evolut-PRO (Medtronic, Irvine, California, United States), and ACURATE neo (Boston Scientific, Natick, Massachusetts, United States) were used. All patients signed informed consent before the procedure and agreed to the anonymous processing of their data (France 2 and France TAVI Registries). The study protocol was developed in accordance with the Declaration of Helsinki and was approved by the France 2 study: 911262.

### Definition of AF

AF was defined as a supraventricular tachyarrhythmia with uncoordinated atrial electrical activation and consequently ineffective atrial contraction. 14 Atrial flutter was defined as a supraventricular tachyarrhythmia with a continuous regular electrical activity, most commonly a saw-tooth pattern on electrocardiogram. 15 Patients with a history of AF or atrial flutter (permanent, persistent, or paroxysmal) or with an AF newly diagnosed during the admission were included in the AF group. Atrial flutter was included in the AF group considering its frequent coexistence with AF. 15

### Antithrombotic Regimen

Unfractionated heparin was used during the procedure to achieve an activated clotting time of 250 to 350 seconds. Heparin was antagonized with 100 IU/kg of protamine at the end of the procedure. Patients without OAC indication received aspirin (75–160 mg) and clopidogrel (300 mg for loading dose and 75 mg/day for maintenance dose) before TAVR, with ongoing dual antiplatelet therapy (DAPT) after the procedure for 3 months. Clopidogrel loading dose was not provided when the patient was under chronic clopidogrel therapy. In patients receiving chronic OAC therapy, clopidogrel was not initiated and OAC and aspirin were continued for 3 months after TAVR. Routinely, OAC therapy was discontinued 5 days prior to the procedure. The choice of OAC was determined at the discretion of the attending physician after the heart team considered the risk for bleeding and thrombotic complications.

### Blood Collections

Whole blood samples were collected by venipuncture the day before and 24 hours following TAVR. PFA-100 (Siemens Healthcare, Marburg, Germany) was used for the CT-ADP assay. Ongoing primary hemostatic disorder was defined by a postprocedural CT-ADP >180 seconds. 9 The extent of P2Y _12_ inhibition by clopidogrel was evaluated 24 hours after TAVR by the analysis of vasodilator-stimulated phosphoprotein (VASP) phosphorylation by flow cytometry as previously described. 16

### Clinical Events

Bleeding complications were assessed according to the Valve Academic Research Consortium-2 (VARC-2) definitions and were classified as follows: life-threatening bleeding, major bleeding, and minor bleeding. Major adverse cardiac and cerebrovascular events (MACCEs) were defined as a composite of all-cause death, myocardial infarction, stroke, and heart failure hospitalization. In-hospital events were collected by careful reviewing of the patient electronic medical records. Patients' follow-up data were obtained through telephone interviews from the patients or their family, the cardiologist, the family physician, or by hospital records.

The primary endpoint of the present analysis was major/life-threatening bleeding complications (MLBCs) at 1 year after TAVR. Secondary endpoint was MACCE at 1 year.

### Statistical Analysis

Categorical variables are represented as frequencies and percentages, and continuous variables are expressed as mean ± standard deviation or median and interquartile values. Differences between two groups were assessed with χ ^2^ tests or Fischer's exact tests for categorical variables. Continuous variables with normal distributions were compared between groups using unpaired Student's *t* -test. The Wilcoxon test was used to analyze continuous variables with skewed distributions. The primary and secondary endpoints were compared with the use of a log-rank test and the hazard ratio (HR), with a 95% confidence interval (CI), was calculated from a Cox proportional hazard model. Factors associated with a *p* -value <0.05 in the univariate analysis were included in the multivariate analysis. In patients with AF, another multivariate analysis was performed, forcing known bleeding risk factors in the model. 17 *p-* Values of <0.05 were considered to indicate statistical significance. All analyses were performed using JMP 13 software (SAS Institute, Cary, North Carolina, United States).

## Results

### Study Population

Among 1,125 consecutive patients undergoing TAVR, 878 patients were enrolled in the present study ( Fig. 1 ). Of these, groups of non-AF and AF included 431 (49%) and 447 (51%) patients, respectively. In the AF group, 46 (10%) had a new-onset AF following TAVR. The cohort was then split into four subgroups according to AF and postprocedural CT-ADP >180 seconds ( Fig. 1 ). Clinical follow-up at 1 year was completed in 873 patients (99.4%).

### Patient Characteristics and Clinical Outcomes in AF and Non-AF Patients

**Fig. 2.**
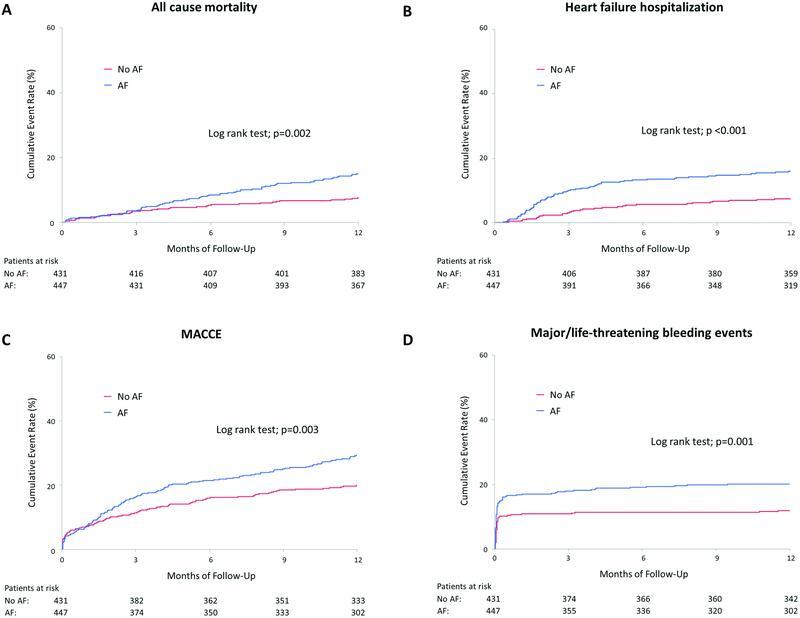
Cumulative incidence of ( **A** ) all-cause mortality, ( **B** ) heart failure hospitalization, ( **C** ) MACCE, and ( **D** ) major/life-threatening bleeding complications were significantly higher in AF patients than in non-AF patients. AF, atrial fibrillation; MACCE, major adverse cardiac and cerebrovascular events.

More comorbidities and higher risk scores were found in patients with AF compared to those without AF ( Supplementary Table S1 ). As expected, AF patients were likely to receive OAC therapy on admission. Preprocedural CT-ADP levels were lower in the AF group, which can partly be explained by the lower mean aortic gradient before TAVR and the lower incidence of clopidogrel therapy on admission. Selections of valves and access site were similar between the two groups ( Supplementary Table S2 ). Lower postprocedural CT-ADP levels, higher platelet reactivity index (PRI)-VASP levels, and lower incidence of clopidogrel use at discharge were observed in AF patients. In contrast, AF patients were likely to receive OAC at discharge. As shown in Fig. 2 , the cumulative event rates of all-cause mortality, heart failure hospitalization, MACCE, and MLBCs at 1 year were significantly higher in AF patients than in non-AF patients.

### Patient Characteristics in Four Groups According to AF and Postprocedural CT-ADP 180 Seconds

In the non-AF group, lower levels of Hb and higher levels of CT-ADP at baseline were evidenced in patients with postprocedural CT-ADP >180 seconds compared to those with postprocedural CT-ADP ≤180 seconds ( Table 1 ). Lower levels of PRI-VASP and Hb after TAVR, and higher incidence of significant PVL were found in patients with postprocedural CT-ADP >180 seconds as well ( Table 2 ). Consistent with the non-AF group, higher CT-ADP levels at baseline and lower PRI-VASP value after TAVR ( Table 2 ) were found in AF patients with postprocedural CT-ADP >180 seconds. In line with this finding, more patients received clopidogrel at discharge and less patients received OAC at discharge in those patients. Lower Hb levels and higher incidence of significant PVL at 1 month after TAVR were also found in patients with postprocedural CT-ADP >180 seconds.

**Table 1:** Baseline clinical characteristics in four groups according to AF and postprocedural CT-ADP 180 seconds

	Non-AF ( *n* = 431)			AF ( *n* = 447)		
	CT-ADP ≤ 180 s ( *n* = 300)	CT-ADP > 180 s ( *n* = 131)	*p* -Value	CT-ADP ≤ 180 s ( *n* = 361)	CT-ADP > 180 s ( *n* = 86)	*p* -Value
Age, y	82 ± 7	82 ± 8	0.90	83 ± 6	84 ± 7	0.82
Men	134 (45)	61 (47)	0.72	172 (48)	43 (50)	0.69
Body mass index, kg/m ^2^	26.9 ± 5.5	26.6 ± 6.0	0.69	27.7 ± 6.3	26.5 ± 4.9	0.17
Logistic EuroSCORE	17.8 ± 11.4	18.0 ± 11.7	0.87	20.1 ± 13.5	23.1 ± 16.7	0.13
EuroSCORE 2	4.9 ± 4.2	4.8 ± 4.7	0.97	5.9 ± 6.5	6.7 ± 8.1	0.33
STS score	5.8 ± 4.6	6.3 ± 5.0	0.41	6.3 ± 5.0	7.7 ± 7.5	0.06
NYHA class III or IV	175/298 (59)	67/130 (52)	0.17	228/359 (64)	61/85 (72)	0.15
Hypertension	250 (83)	109 (83)	0.97	301 (83)	79 (92)	0.048
Dyslipidemia	187 (62)	80 (61)	0.80	213 (59)	56 (65)	0.30
Diabetes mellitus	102 (34)	48 (37)	0.60	107 (30)	34 (40)	0.08
CKD (eGFR <60 mL/min/1.73 m ^2^ )	153/299 (51)	75/131 (57)	0.24	238 (66)	59 (69)	0.64
Hemodialysis	5 (2)	4 (3)	0.46	4 (1)	4 (5)	0.048
Prior myocardial infarction	37 (12)	13 (10)	0.47	44 (12)	14 (16)	0.31
Prior PCI	102 (34)	42 (32)	0.69	112 (31)	35 (41)	0.09
Prior CABG	35 (12)	13 (10)	0.60	49 (11)	10 (12)	0.83
Peripheral artery disease	75 (25)	28 (21)	0.41	117 (32)	22 (26)	0.22
Prior stroke	48 (16)	13 (10)	0.10	55 (15)	16 (19)	0.44
COPD	43 (14)	16 (12)	0.56	63 (17)	10 (12)	0.19
CHA2DS2VASC score	–	–	–	4.7 ± 1.3	4.8 ± 1.5	0.36
Medication at baseline						
DAPT	96 (32)	48 (37)	0.35	43 (12)	19 (22)	0.01
Clopidogrel	92 (31)	43 (33)	0.66	50 (14)	19 (22)	0.06
OAC	18 (6)	2 (2)	0.04	287 (80)	50 (58)	<0.001
VKA	12 (4)	1 (1)	0.12	195 (54)	33 (38)	0.01
DOAC	6 (2)	1 (1)	0.68	92 (25)	17 (20)	0.27
Echocardiography data at baseline						
Mean aortic gradient, mmHg	49 ± 15	48 ± 14	0.65	45 ± 12	44 ± 12	0.63
LVEF, %	53 ± 16	54 ± 14	0.68	52 ± 15	53 ± 16	0.63
LVEDD, mm	50 ± 8	50 ± 8	0.93	50 ± 8	50 ± 8	0.92
LVESD, mm	34 ± 9	35 ± 9	0.64	35 ± 10	34 ± 9	0.36
Types of aortic stenosis						
High-gradient	255/298 (86)	109 (83)	0.53	287 (80)	69 (80)	0.88
Low-flow, low-gradient with reduced ejection fraction	31/298 (10)	17 (13)	0.44	48 (13)	7 (8)	0.19
Low-flow, low-gradient with preserved ejection fraction	4/298 (1)	3 (2)	0.48	20 (6)	6 (7)	0.61
Laboratory tests at baseline						
WBC, 10 ^3^ /mm ^3^	7.5 ± 2.2	7.2 ± 2.1	0.09	7.6 ± 2.1	6.9 ± 2.0	0.004
Hb, g/dL	12.4 ± 1.7	11.8 ± 1.5	<0.001	12.2 ± 1.7	11.5 ± 1.9	<0.001
Platelet count, 10 ^3^ /mm ^3^	235 ± 76	223 ± 61	0.11	233 ± 72	201 ± 55	<0.001
CRP, mg/dL	0.4 (0.4–0.8)	0.4 (0.4–0.6)	0.29	0.4 (0.4–1.1)	0.5 (0.4–1.6)	0.19
Creatinine, µmol/L	88 (72–119)	95 (77–121)	0.16	103 (82–134)	123 (88–149)	0.07
eGFR, mL/min/1.73 m ^2^	59 ± 21	56 ± 22	0.13	52 ± 19	48 ± 21	0.09
CT-ADP, s	193 ± 76	243 ± 72	<0.001	161 ± 68	212 ± 77	<0.001

Abbreviations: AF, atrial fibrillation; CABG, coronary artery bypass grafting; CKD, chronic kidney disease; COPD, chronic obstructive pulmonary disease; CRP, C-reactive protein; CT-ADP, closure time of adenosine diphosphate; DAPT, dual antiplatelet therapy; DOAC, direct oral anticoagulant; eGFR, estimated glomerular filtration rate; Hb, hemoglobin; LVEDD, left ventricular end-diastolic diameter; LVEF, left ventricular ejection fraction; LVESD, left ventricular end-systolic diameter; NYHA, New York Heart Association; OAC, oral anticoagulant; PCI, percutaneous coronary intervention; VKA, vitamin K antagonist; WBC, white blood cell.

Note: Values are *n* (%) or *n* / *N* (%), mean ± SD, or median (interquartile range).

**Table 2:** Procedural and postprocedural characteristics in four groups according to AF and postprocedural CT-ADP 180 seconds

	Non-AF ( *n* = 431)			AF ( *n* = 447)		
	CT-ADP ≤ 180 s ( *n* = 300)	CT-ADP > 180 s ( *n* = 131)	*p* -Value	CT-ADP ≤ 180 s ( *n* = 361)	CT-ADP > 180 s ( *n* = 86)	*p* -Value
Procedural characteristics						
Femoral approach	265/299 (89)	122/131 (93)	0.15	322 (92)	81 (94)	0.49
Prothesis type						
Balloon-expandable	178 (59)	78 (60)	0.97	219 (61)	55 (64)	0.57
Self-expandable	122 (41)	53 (40)	0.97	142 (39)	31 (36)	0.57
Prosthesis diameter, mm	27 ± 3	26 ± 3	0.67	27 ± 3	26 ± 3	0.07
Sheath diameter, mm	15 ± 2	15 ± 2	0.53	15 ± 2	15 ± 2	0.90
Postprocedural characteristics						
24 hours after TAVR						
CT-ADP, sec	116 ± 28	278 ± 39	<0.001	112 ± 28	268 ± 43	<0.001
PRI-VASP, %	67 ± 15	57 ± 20	<0.001	73 ± 14	67 ± 19	0.007
At discharge						
WBC, 10 ^3^ /mm ^3^	7.8 ± 2.7	7.9 ± 4.0	0.85	7.9 ± 2.7	7.3 ± 2.4	0.06
Hb, g/dL	10.4 ± 1.4	9.9 ± 1.4	<0.001	10.3 ± 1.4	9.9 ± 1.2	0.01
CRP, mg/dL	1.0 (1.0–1.1)	1.1 (1.0–1.1)	0.52	1.1 (1.0–1.1)	1.1 (1.0–1.1)	0.44
Creatinine, µmol/L	54 (28–90)	48 (26–85)	0.09	57 (33–94)	48 (25–86)	0.12
Medication at discharge						
Aspirin	292/299 (98)	129/131 (98)	0.73	337 (93)	78 (91)	0.39
Clopidogrel	259/299 (87)	122/131 (93)	0.05	68 (19)	35 (41)	<0.001
OAC	21/299 (7)	5/131 (4)	0.20	327 (91)	58 (67)	<0.001
VKA	12/299 (4)	1/131 (1)	0.12	194 (54)	39 (45)	0.19
DOAC	9/299 (3)	4/131 (3)	1.00	133 (37)	19 (22)	0.01
Number of antithrombotic therapies			0.30			0.03
0	3/299 (1)	1/131 (1)		0 (0)	1 (1)	
1	20/299 (7)	5/131 (4)		16 (4)	8 (9)	
2	276/299 (92)	124/131 (95)		319 (88)	68 (79)	
3	0/299 (0)	1/131 (1)		26 (7)	9 (10)	
Echocardiography at 1 month ( *n* = 820)						
LVEF, %	59 ± 12	61 ± 11	0.15	56 ± 12	58 ± 10	0.19
LVEDD, mm	50 ± 8	50 ± 7	0.67	51 ± 8	52 ± 7	0.60
LVESD, mm	33 ± 9	32 ± 8	0.26	35 ± 10	35 ± 8	0.65
Mean prosthetic valve gradient, mmHg	11 ± 5	11 ± 7	0.23	9 ± 4	10 ± 5	0.10
Significant PVL (> mild)	20/285 (7)	18/120 (15)	0.01	21/335 (6)	19/81 (23)	<0.001

Abbreviations: AF, atrial fibrillation; CRP, C-reactive protein; CT-ADP, closure time of adenosine diphosphate; Hb, hemoglobin; DOAC, direct oral anticoagulant; LVEDD, left ventricular end-diastolic diameter; LVEF, left ventricular ejection fraction; LVESD, left ventricular end-systolic diameter; OAC, oral anticoagulant; PRI-VASP, platelet reactivity index by vasodilator-stimulated phosphoprotein; PVL, paravalvular leak; TAVR, transcatheter aortic valve replacement; VKA, vitamin K antagonist; WBC, white blood cell.

Note: Values are *n* (%) or *n* / *N* (%), mean ± SD, or median (interquartile range).

### Clinical Outcomes in Four Groups According to AF and Postprocedural CT-ADP 180 seconds

**Fig. 3.**
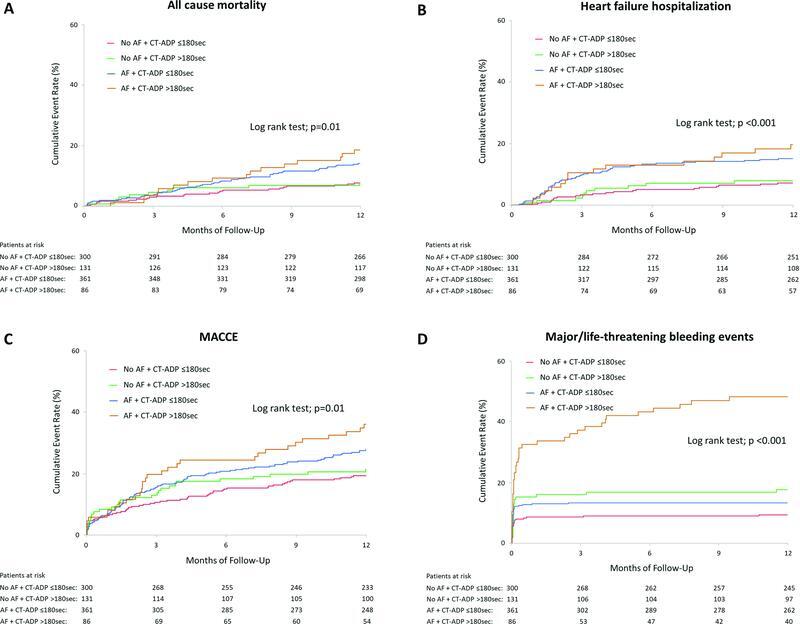
Cumulative event rates of all-cause mortality ( **A** ), heart failure hospitalization ( **B** ), MACCE ( **C** ), and major/life-threatening bleeding events ( **D** ) in four groups according to AF and postprocedural CT-ADP >180 seconds. A drastic increase of bleeding events was evidenced in patients with AF + CT-ADP >180 seconds. AF, atrial fibrillation; CT-ADP, closure time adenosine diphosphate; MACCE, major adverse cardiac and cerebrovascular events.

Among non-AF patients, postprocedural CT-ADP >180 seconds was associated with increased MLBCs during hospitalization, at 1 year, and at 2 year after TAVR ( Supplementary Table S3 ). Likewise, the impact of postprocedural CT-ADP >180 seconds on in-hospital, 1-year, and 2-year MLBCs was evidenced in AF patients ( Supplementary Table S3 ). Kaplan–Meier curves in Fig. 3 clarify that the patients with AF and postprocedural CT-ADP >180 seconds had an increased rate of cumulative adverse events, remarkably in MLBCs, compared to the other three groups. Consistently, Kaplan–Meier curves in Supplementary Fig. S1 demonstrate an increased cumulative bleeding event in patients with prior OAC and postprocedural CT-ADP >180 seconds, whereas no additional impact of prior DAPT over postprocedural CT-ADP >180 seconds was found.

### Predictors of MLBCs in AF and Non-AF Patients

In non-AF patients, prior myocardial infarction, mean aortic gradient, Hb levels, and platelet count were independent predictors of MLBCs at 1 year, whereas postprocedural CT-ADP >180 seconds turned out to be insignificant after the adjustment ( Supplementary Table S4 ). In contrast to the non-AF cohort, the multivariate analysis demonstrates that postprocedural CT-ADP >180 seconds was significantly associated with MLBCs at 1 year in AF patients ( Supplementary Table S5 , Model 1). Known bleeding risk factors in AF patients including the components of HASBLED score 17 and OAC administration were forced in Supplementary Table S5 , Model 2, reinforcing the impact of postprocedural CT-ADP >180 seconds on MLBCs at 1 year.

### Predictors of MLBCs and MACCE in the Whole Cohort

**Fig. 4.**
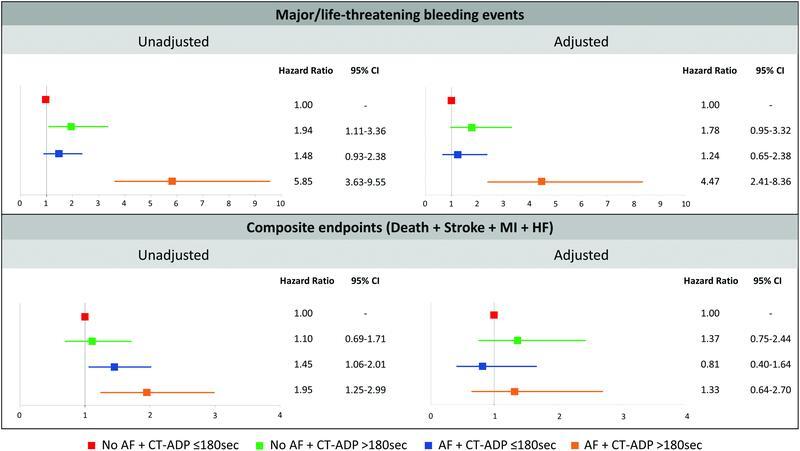
Multivariate Cox regression analyses illustrate that patients with AF and ongoing primary hemostatic disorder (postprocedural CT-ADP >180 seconds) were associated with an increased risk of major/life-threatening bleeding complications at follow-up, whereas no impact was evidenced in MACCE. CT-ADP, closure time adenosine diphosphate; MACCE, major adverse cardiac and cerebrovascular events.

After adjustment of clinical variables, Hb levels at baseline and AF + postprocedural CT-ADP >180 seconds remained independent predictors of 1-year MLBCs ( Fig. 4 and Supplementary Table S6 ). For 1-year MACCE, elevated CRP levels and a nonfemoral approach were predictive factors, whereas AF + CT-ADP >180 seconds was no longer associated with MACCE after the adjustment ( Fig. 4 and Supplementary Table S7 ).

### Late Bleeding Events Following TAVR

**Fig. 5.**
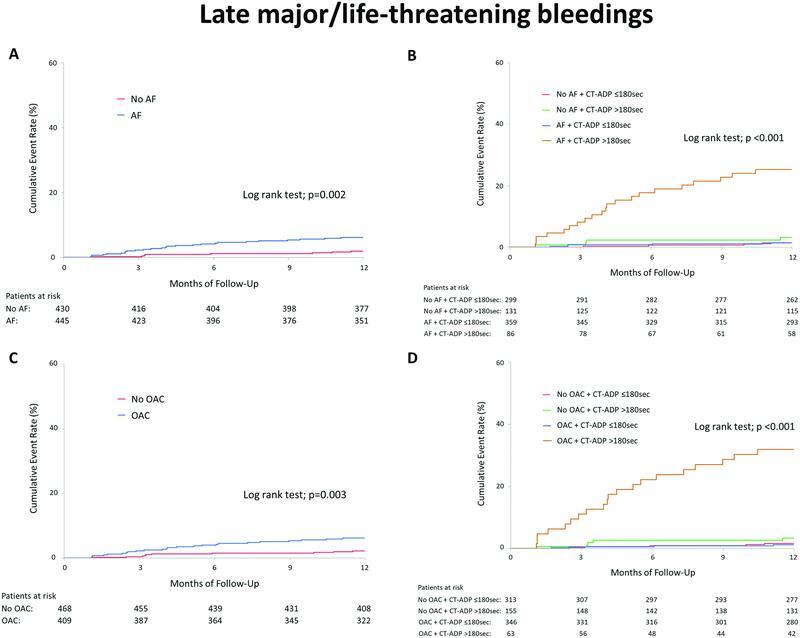
Cumulative event rates of late major/life-threatening bleeding events (>30 days after TAVR) in AF and non-AF patients ( **A** ), in four groups according to AF and postprocedural CT-ADP >180 seconds ( **B** ), in patients with and without OAC ( **C** ), and in four groups according to the use of OAC at discharge and postprocedural CT-ADP >180 seconds ( **D** ). AF, atrial fibrillation; CT-ADP, closure time adenosine diphosphate; OAC, oral anticoagulant; TAVR, transcatheter aortic valve replacement.

Fig 5 illustrates the cumulative event rates of late bleeding events (>30 days after TAVR). AF patients were prone to late MLBCs compared to non-AF patients ( Fig. 5A ), which was mainly driven by the patients with postprocedural CT-ADP >180 seconds ( Fig. 5B ). Fig. 5(C, D) emphasizes that the late bleeding events in patients with postprocedural CT-ADP >180 seconds were mainly due to continuous OAC therapy after TAVR. On the other hand, an additional impact of continuous DAPT over ongoing primary hemostasis disorder on late bleeding events was not clarified ( Supplementary Fig. S2 ). Gastrointestinal bleeding was the most frequent type of late MLBCs (62%), followed by intracranial and nose or mouth bleedings ( Supplementary Table S8 ).

### Patients with and without Clopidogrel at Discharge

Given the strong relationship between the use of clopidogrel and prolonged CT-ADP levels ( Table 2 ), a sensitivity analysis was performed with patients with and without clopidogrel at discharge. As shown in Supplementary Fig. S3 , the deleterious effects of prolonged CT-ADP levels (>180 seconds) in AF patients were consistently observed in patients with and without clopidogrel at discharge. Although the magnitude of AF + postprocedural CT-ADP >180 seconds in predicting 1-year MLBCs was higher in patients without clopidogrel at discharge (HR: 4.40; 95% CI: 2.66–7.15) compared to patients with clopidogrel at discharge (HR: 3.41; 95% CI: 1.50–8.21), the HRs were not statistically different between the two cohorts ( *p*_interaction_ = 0.59).

## Discussion

The principal findings of this study are summarized as follows: first, patients with AF after TAVR were associated with a higher rate of long-term adverse clinical events including bleedings as compared with non-AF patients. Second, postprocedural CT-ADP >180 seconds was a major determinant of MLBCs in AF patients but not in non-AF patients. Third, AF + postprocedural CT-ADP >180 seconds was strongly associated with late bleeding events after TAVR. These findings suggest that ongoing primary hemostatic disorders as evaluated by CT-ADP could be a reliable predictor of long-term bleeding events particularly in TAVR patients with AF.

### Atrial Fibrillation in TAVR Patients

While the reported incidence of pre-existing AF in TAVR patients ranges from 16 to 51%, 5 6 18 data on new-onset AF are more scattered compared with pre-existing AF, being reported between 1 and 32%. 6 19 In line with these findings, pre-existing or new-onset AF was found in 51% of our cohort.

Although previous investigations have shown conflicting results concerning the relationship between AF and bleeding events, 6 a large registry including 72,660 patients who underwent non-apical TAVR clarified that both pre-existing and new-onset AF were associated with increased risk of bleeding events. 19 The bleeding diathesis in AF patients can be partly explained by the comorbidities and multiple antithrombotic therapy. 6 19 20 Moreover, a recent report by Lother et al delineated that the incidence of bleeding defines the outcome of patients with AF after transfemoral TAVR, 5 indicating that particular care in bleeding prevention is indispensable for TAVR patients with AF. Our current study highlights the high-risk profile of AF patients treated with multiple antithrombotic therapies following TAVR, which may have led to an increased risk of MLBCs at follow-up.

### Primary Hemostatic Disorder in TAVR Patients

Among various factors that could favor bleeding events following TAVR, special attention was given to the role played by primary hemostatic disorders. CT-ADP is a primary hemostasis point-of-care test which can be evaluated by the PFA-100. 21 The system aspirates citrated whole blood at high shear rates through disposable cartridges containing an aperture within a membrane coated with collagen and ADP which serves as a platelet-stimulating agent. Studies have indicated that the clinical performance of CT-ADP is excellent in both inherited and acquired von Willebrand disease (vWD). 9 22 Importantly, the value will not be influenced by aspirin or OACs.

Severe AS induces a very turbulent blood flow at the vicinity of the aortic valve, causing high shear stress that promotes deployment and cleavage of von Willebrand factor (VWF)'s high-molecular-weight (HMW)-multimers results in an increase of CT-ADP. 23 Moreover, a significant PVL (>mild) can also induce HMW-multimer defects because of its turbulent flow. Although postprocedural CT-ADP >180 seconds has been predictive of the presence of significant PVL with early generation TAVR valves, 9 our data demonstrate a stepwise decrease of significant PVL with newer valves across the last 5 years, whereas the prevalence of patients with postprocedural CT-ADP >180 seconds holds more than 20% ( Supplementary Fig. S4 ). This result suggests that the mild relationship between significant PVL and increased CT-ADP levels in the current TAVR era may have mitigated the impact of CT-ADP >180 seconds on adverse cardiac events ( Fig. 4 ). 24 Several mechanisms could be responsible for the discrepancy between significant PVL and postprocedural CT-ADP levels; first, mild PVL may also have an impact on CT-ADP levels. Our data demonstrate a higher incidence of postprocedural CT-ADP >180 seconds in patients with mild PVL compared to those without PVL at discharge (29 vs. 19%) and at 1-month follow-up (28 vs. 19%), suggesting that the defects of VWF HMW-multimers may also be induced by mild PVL. Second, high shear stress-induced VWF HMW-multimer defects can be created by other types of valvular heart disease 23 25 which were not fully evaluated in our cohort. Third, the CT-ADP may also predict the bleeding tendency resulting from functional platelet alteration in patients with defects other than vWD. 22 Taken together, postprocedural CT-ADP levels may exhibit persistent platelet dysfunction which can mainly affect AF patients who are likely to have multiple comorbidities and receive OACs after TAVR.

### Antithrombotic Therapy in TAVR Patients

While prior guidelines on antithrombotic therapy in TAVR patients have been mostly based on expert opinion, latest insights were provided by recent randomized trials. The GALILEO trial 26 randomized patients without an indication of OAC to low-dose rivaroxaban plus aspirin for 3 months, followed by rivaroxaban alone versus aspirin plus clopidogrel for 3 months, followed by aspirin alone. The trial was terminated because of 69% relative increase in mortality and 50% increase in MLBCs in the rivaroxaban arm. In POPULAR TAVI trial cohort B, 20 patients undergoing TAVR with an indication of OAC (mainly AF 94.9%) were randomized to receive either OAC alone or OAC plus clopidogrel for 3 months. The incidence of serious bleeding was lower with OAC alone than with OAC plus clopidogrel. Altogether, these two trials reinforced the detrimental effects of OAC when it is combined with antiplatelet therapies. Given these results, the 2021 ESC/EACTS (European Society of Cardiology/European Association for Cardio-Thoracic Surgery) guidelines recommend single OAC following TAVR in patients with an indication of OAC and single antiplatelet therapies (SAPTs) in patients without indication of OAC. 13 In addition, a recent consensus document from the ESC Working Group proposes OAC plus SAPT for patients with an indication of OAC when PCI has been performed within 3 months. 27 Although a tailored antithrombotic therapy is recommended in those patients according to the bleeding diathesis, an indicator of future bleeding events is lacking and an optimal dose of OAC remains unclear.

Although the noxious impact of OAC in addition to antiplatelet therapy on bleeding events was evidenced in recent trials, the extent of platelet functional defect has not been explored. We previously described the extent of platelet inhibition by clopidogrel in TAVR patients, showing that an appropriate response to clopidogrel (PRI-VASP ≤50%) was contributed to major bleeding events. 28 In contrast, CT-ADP reflects a broader spectrum of platelet function, including defects of VWF HMW-multimers, various platelet disorders, and platelet inhibition by P2Y _12_ inhibitors. 7 22 On a pragmatical approach, our present data clearly suggest that the inhibition of secondary hemostasis by OAC over primary hemostatic disorder may hamper the hemostasis process extensively, resulting in increasing the prevalence of bleeding complications. Therefore, a reduced amount of OAC may be favorable for patients with prolonged CT-ADP levels and a shorter duration of dual antithrombotic therapy can be considered in patients with recent coronary stenting.

It is noteworthy that the latest evidence from ENVISAGE-TAVI AF trial 29 showed a higher incidence of major bleeding events with edoxaban than with VKA in AF patients who underwent TAVR. Interestingly, patients who received low edoxaban dose (30 mg once daily) had similar incidence of major bleeding to those who received VKA, suggesting that that a lower dose of DOAC may mitigate the late bleeding risk after TAVR. In the latest ESC/EACTS guidelines for the management of AF, 14 a reduced dose of DOAC is recommended based on the bleeding risk profile of AF patients. While our study emphasizes that ongoing primary hemostatic disorder, evaluated by postprocedural CT-ADP, may also be a predictor of future bleeding events in TAVR patients with AF, further trials are required to elucidate whether similar dose reduction can be safely applied to those patients.

### Study Limitations

We acknowledge the following limitations: first, the analyses were performed on the basis of a single center data set with uncertain generalizability. Second, CT-ADP prolongation could be influenced by the presence of low Hb levels, low platelet count, and P2Y _12_ inhibitors. Nevertheless, the noxious impact of CT-ADP >180 seconds on bleeding events was confirmed by adjusting the Hb levels and platelet count in the multivariate analysis and the sensitivity analysis with and without clopidogrel at discharge. Third, assessments of CT-ADP were only performed 24 hours after TAVR and were not repeated during the follow-up. Forth, since the majority of patients received dual antithrombotic therapy at discharge (90%) ( Supplementary Table S9 ), we were not able to investigate the relationship between the number of antithrombotic therapies and bleeding events. Fifth, the antithrombotic regimen at the time of the events was not captured. Sixth, the HASBLED score, which is an established bleeding risk score, was not collected in our registry. Thus, the classical bleeding risk in AF patients with and without CT-ADP >180 seconds remains uncertain. However, several components of the score were available in our study, showing no difference between the two groups. Seventh, certain blood parameters such as serum iron concentration 30 or red blood cell distribution 31 are associated with bleeding complications following TAVR but were not collected in our registry. Eighth, multivariate analysis should be interpreted with caution because of the limited sample size and the nature of observational study. There are inherent limitations in this type of study, mainly related to unknown confounding factors. Thus, further investigations are required to validate the CT-ADP-guided care after TAVR. 32

## Conclusion

Ongoing primary hemostatic disorder, evaluated by postprocedural CT-ADP, was independently associated with MLBCs among TAVR patients with AF. A large majority of late MLBCs occurred in patients with OAC and postprocedural CT-ADP >180 seconds. These findings indicate that a better individualized and risk-adjusted antithrombotic therapy may be considered for reducing bleeding events in TAVR patients with prolonged CT-ADP levels.

## Notes

**What Is Known about This Topic?**

- A tailored antithrombotic therapy in transcatheter aortic valve replacement (TAVR) patients is needed for atrial fibrillation (AF) patients according to the bleeding diathesis, but an indicator of future bleeding events is lacking.
- In TAVR patients, prolonged closure time of adenosine diphosphate (CT-ADP) in AF patients was associated with major/life-threatening bleeding complications at follow-up.
- Likewise, an increased bleeding risk was found in patients with oral anticoagulant (OAC) and prolonged CT-ADP.

**What Does This Paper Add?**

- Our present data suggest that the inhibition of secondary hemostasis by OAC over primary hemostatic disorder may hamper the hemostasis process extensively, resulting in increasing the prevalence of bleeding complications.
- A tailored and risk-adjusted antithrombotic therapy after TAVR may be considered particularly in AF patients with primary hemostatic disorder.
