# Supplementary material for: Combination of Primary Hemostatic Disorders and Atrial Fibrillation Increases Bleeding Events Following Transcatheter Aortic Valve Replacement
Source: TH Open. 2023 May 11;7(2):e117–27. doi: 10.1055/a-2068-5783 (PMC10174752; doi:10.1055/a-2068-5783)
Supplement: Supplementary file 1 — Supplementary Material [file 10-1055-a-2068-5783-s23020007.pdf]

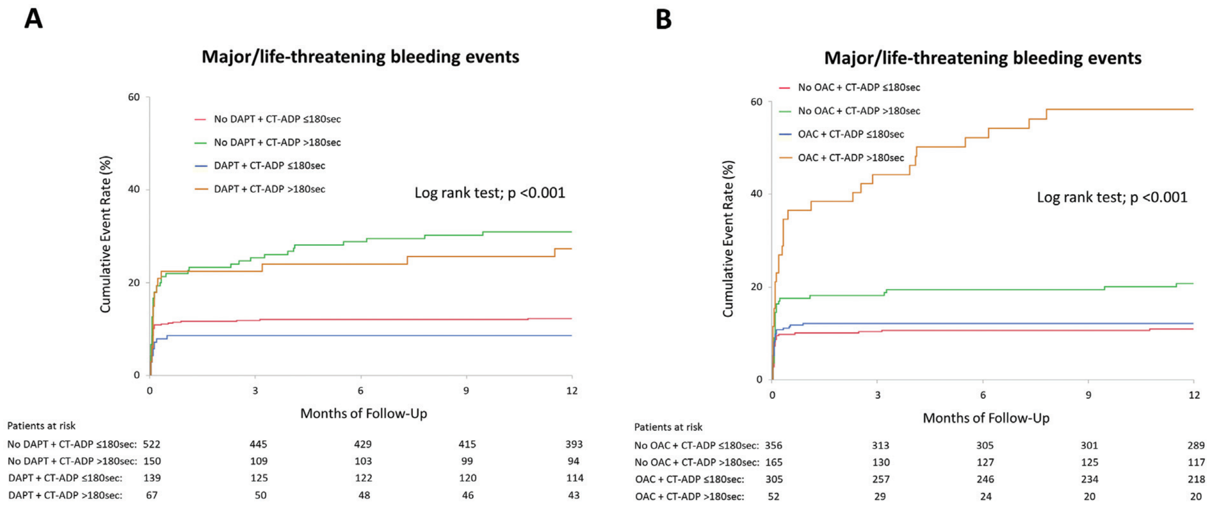

Supplementary Fig. S1 Cumulative incidence of bleeding events according to preprocedural antithrombotic therapies and postprocedural CT-ADP >180 seconds.

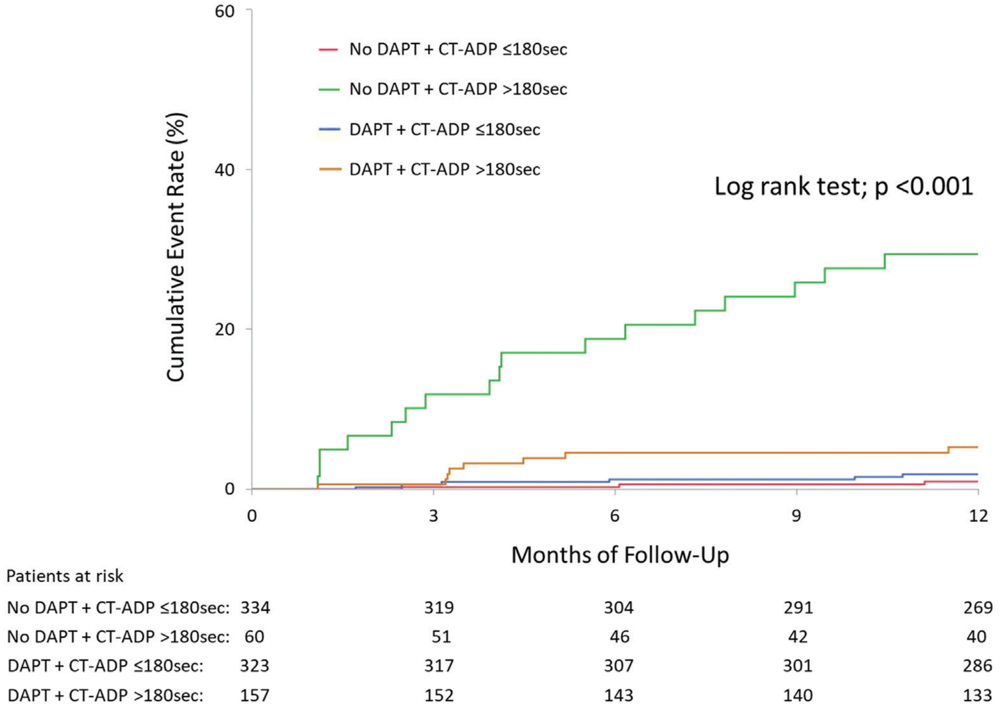

Supplementary Fig. S2 Cumulative incidence of late bleeding events according to postprocedural DAPT and postprocedural CT-ADP >180 seconds. DAPT, dual antiplatelet therapy.

 © 2023. The Author(s).

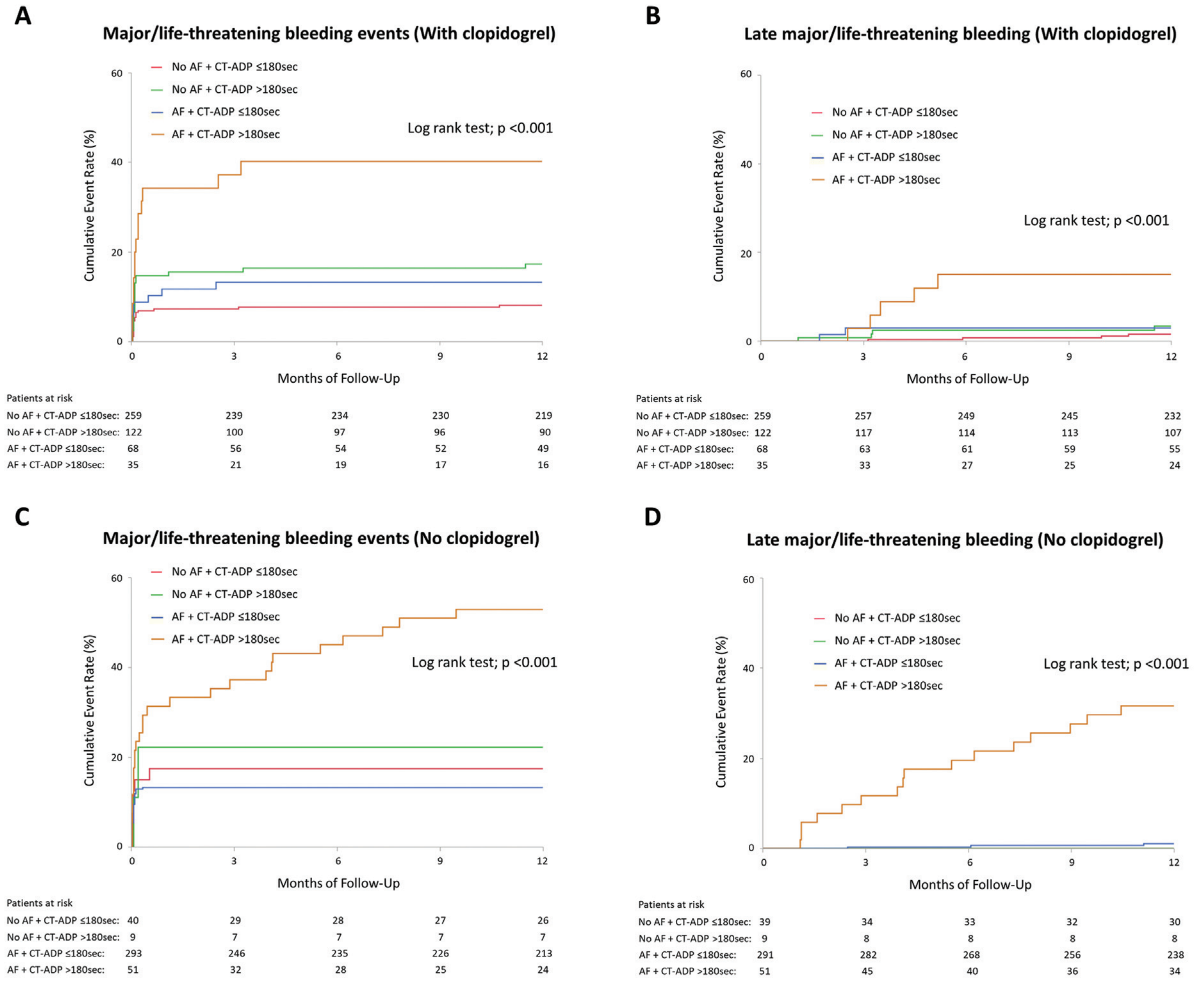

Supplementary Fig. S3 Cumulative incidence of bleeding events in patients with and without clopidogrel at discharge.

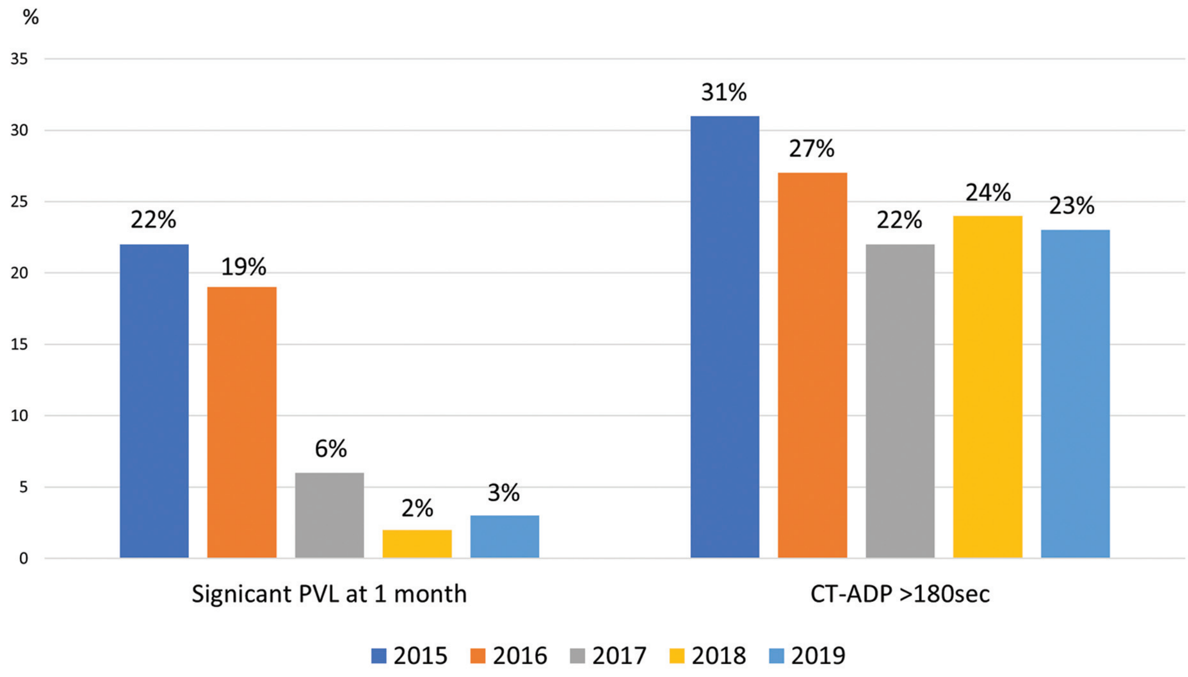

Supplementary Fig. S4 Incidence of PVL and postprocedural CT-ADP >180 seconds between 2015 and 2019. PVL, paravalvular leak.

 © 2023. The Author(s).

Supplementary Table S1 Baseline characteristics of patients with and without atrial fibrillation

	Non-AF ($n=431$)	AF ($n=447$)	p-Value
Age, y	82 ± 7	83 ± 6	0.006
Men	195 (45)	215 (48)	0.40
Body mass index, kg/m^2	26.8 ± 5.7	27.4 ± 6.1	0.17
Logistic EuroSCORE	17.9 ± 11.5	20.7 ± 14.2	0.006
EuroSCORE 2	4.9 ± 4.4	6.1 ± 6.8	0.005
STS score	6.0 ± 4.7	6.6 ± 5.6	0.11
NYHA class III or IV	242/428 (57)	289/444 (65)	0.01
Hypertension	359 (83)	380 (85)	0.49
Dyslipidemia	267 (62)	269 (60)	0.59
Diabetes mellitus	150 (35)	141 (32)	0.31
CKD (eGFR <60 mL/min/1.73 m^2)	228/430 (53)	297/447 (66)	<0.001
Hemodialysis	9 (2)	8 (2)	0.75
Prior myocardial infarction	50 (12)	58 (13)	0.54
Prior PCI	144 (33)	147 (33)	0.87
Prior CABG	48 (11)	49 (11)	0.93
Peripheral artery disease	103 (24)	139 (31)	0.02
Prior stroke	81 (14)	71 (16)	0.47
COPD	59 (14)	73 (16)	0.27
Medication at baseline			
DAPT	144 (33)	62 (14)	<0.001
Clopidogrel	13 (31)	69 (15)	<0.001
OAC	20 (5)	337 (77)	<0.001
VKA	13 (3)	228 (51)	<0.001
DOAC	7 (2)	109 (24)	<0.001
Echocardiography data at baseline			
Mean aortic gradient, mmHg	48.6 ± 14.8	44.7 ± 12.3	<0.001
LVEF, %	53 ± 15	52 ± 15	0.28
LVEDD, mm	50 ± 8	50 ± 8	0.75
LVESD, mm	35 ± 9	35 ± 10	0.48
Laboratory tests at baseline			
WBC, 10^3/mm^3	7.4 ± 2.1	7.5 ± 2.1	0.76
Hb, g/dL	12.2 ± 1.7	12.1 ± 1.7	0.34
Platelet count, 10^3 /mm^3	231 ± 72	227 ± 70	0.35
CRP, mg/dL	0.4 (0.4–0.77)	0.4 (0.4–1.2)	0.002
Creatinine, μmol/L	90 (73–120)	103 (83–138)	<0.001
eGFR, mL/min/1.73 m^2	58 ± 22	51 ± 20	<0.001
CT-ADP, s	208 ± 78	171 ± 73	<0.001

Abbreviations: AF, atrial fibrillation; CABG, coronary artery bypass grafting; CKD, chronic kidney disease; COPD, chronic obstructive pulmonary disease; CRP, C-reactive protein; CT-ADP, closure time of adenosine diphosphate; DAPT, dual antiplatelet therapy; DOAC, direct oral anticoagulant; eGFR, estimated glomerular filtration rate; Hb, hemoglobin; LVEDD, left ventricular end-diastolic diameter; LVEF, left ventricular ejection fraction; LVESD, left ventricular end-systolic diameter; NYHA, New York Heart Association; OAC, oral anticoagulant; PCI, percutaneous coronary intervention; VKA, vitamin K antagonist; WBC, white blood cell.
Note: Values are n (%) or n/N (%), mean ± SD, or median (interquartile range).

 © 2023. The Author(s).

Supplementary Table S2 Procedural and postprocedural characteristics of patients with and without atrial fibrillation

	Non-AF ($n=431$)	AF ($n=447$)	p-Value
Procedural characteristics			
Femoral approach	387/430 (90)	413/447 (92)	0.21
Prothesis type			
Balloon-expandable	256 (59)	274 (61)	0.56
Self-expandable	175 (41)	173 (39)	0.56
Prosthesis diameter, mm	26 ± 3	27 ± 3	0.18
Sheath diameter, mm	15 ± 2	15 ± 2	0.82
Postprocedural characteristics			
24 hours after TAVR			
CT-ADP, s	165 ± 81	142 ± 69	<0.001
CT-ADP >180 s	131 (30)	86 (19)	<0.001
PRI-VASP, %	64 ± 17	72 ± 15	<0.001
At discharge			
WBC, 10^3 /mm^3	7.8 ± 3.1	7.8 ± 2.7	0.89
Hb, g/dL	10.3 ± 1.4	10.2 ± 1.4	0.72
CRP, mg/dL	1.0 (1.0–1.1)	1.1 (1.0–1.1)	0.04
Creatinine, μmol/L	52 (28–89)	55 (32–91)	0.19
Medication at discharge			
Aspirin	421/430 (98)	415/447 (93)	<0.001
Clopidogrel	381/430 (89)	103/447 (23)	<0.001
OAC	26/430 (6)	385/447 (86)	<0.001
VKA	13/430 (3)	233/447 (52)	<0.001
DOAC	13/430 (3)	152/447 (34)	<0.001
Number of antithrombotic therapies			<0.001
0	4/430 (1)	1/447 (0.2)	
1	25/430 (6)	24/447 (5)	
2	400/430 (93)	387/447 (87)	
3	1/430 (0.2)	35/447 (8)	
Echocardiography at 1 month ($n=820$)			
LVEF, %	60 ± 12	56 ± 12	<0.001
LVEDD, mm	50 ± 8	51 ± 7	0.07
LVESD, mm	33 ± 9	35 ± 9	0.007
Mean prosthetic valve gradient, mmHg	11 ± 6	10 ± 5	<0.001
Significant PVL (> mild)	38/405 (9)	40/416 (10)	0.91

Abbreviations: AF, atrial fibrillation; CRP, C-reactive protein; CT-ADP, closure time of adenosine diphosphate; Hb, hemoglobin; DOAC, direct oral anticoagulant; LVEDD, left ventricular end-diastolic diameter; LVEF, left ventricular ejection fraction; LVESD, left ventricular end-systolic diameter; OAC, oral anticoagulant; PRI-VASP, platelet reactivity index by vasodilator-stimulated phosphoprotein; PVL, paravalvular leak; TAVR, transcatheter aortic valve replacement; VKA, vitamin K antagonist; WBC, white blood cell.
Note: Values are n (%) or n/N (%), mean ± SD, or median (interquartile range).

 © 2023. The Author(s).

Supplementary Table S3 Clinical events following transcatheter aortic valve replacement

	Non-AF ($n = 431$)			AF ($n = 447$)		
	CT-ADP ≤180 s ($n = 300$)	CT-ADP >180 s ($n = 131$)	*p*-Value	CT-ADP ≤180 s ($n = 361$)	CT-ADP >180 s ($n = 86$)	*p*-Value
In-hospital events ($n = 878$)						
Major vascular complication	19 (6)	12 (9)	0.30	33 (9)	13 (15)	0.10
Annulus rupture	0 (0)	0 (0)	–	0 (0)	0 (0)	–
Coronary obstruction	2 (1)	0 (0)	0.35	1 (0.3)	0 (0)	1.00
Conversion to open heart surgery	0 (0)	0 (0)	–	0 (0)	0 (0)	–
Pericardial effusion	2 (1)	0 (0)	0.35	4 (1)	3 (3)	0.13
Major/life-threatening bleeding	26 (9)	20 (15)	0.04	51 (14)	30 (35)	<0.001
Major bleeding	19 (6)	16 (12)	0.04	33 (9)	21 (24)	<0.001
Life-threatening bleeding	8 (3)	6 (5)	0.38	19 (5)	9 (10)	0.07
Minor bleeding	66 (22)	27 (21)	0.75	60 (17)	17 (20)	0.49
Transfusion ≥2 units	40 (13)	24 (18)	0.18	58 (16)	28 (33)	<0.001
1-year outcome ($n = 873$)						
All cause death	25/298 (8)	10/130 (8)	0.81	51/359 (14)	16/86 (19)	0.31
Cardiovascular death	20/298 (7)	7/130 (5)	0.60	20/358 (6)	5/85 (6)	1.00
Cerebrovascular event	20/298 (7)	12/130 (9)	0.36	17/359 (5)	6/86 (7)	0.42
Myocardial infarction	5/298 (2)	3/130 (2)	0.70	4/359 (1)	2/86 (2)	0.33
Heart failure hospitalization	21/298 (7)	10/130 (8)	0.81	53/359 (15)	16/86 (19)	0.38
MACCE	59/298 (20)	28/130 (22)	0.68	100/359 (28)	31/86 (36)	0.13
Major/life-threatening bleeding	28/298 (9)	23/130 (18)	0.01	47/359 (13)	41/86 (48)	<0.001
2-year outcome ($n = 664$)						
All cause death	42/228 (18)	15/92 (16)	0.65	93/279 (33)	22/65 (34)	0.94
Cardiovascular death	26/228 (11)	10/92 (11)	0.89	37/278 (13)	7/64 (11)	0.61
Cerebrovascular event	17/228 (7)	8/92 (9)	0.71	21/279 (8)	5/65 (8)	0.96
Myocardial infarction	8/228 (4)	3/92 (3)	1.00	2/279 (1)	2/65 (3)	0.16
Heart failure hospitalization	30/228 (13)	13/92 (14)	0.82	60/279 (22)	17/65 (26)	0.42
MACCE	77/228 (34)	31/92 (31)	0.99	128/279 (46)	34/65 (52)	0.35
Major/life-threatening bleeding	26/228 (11)	21/92 (23)	0.009	45/279 (16)	35/65 (54)	<0.001

Abbreviations: AF, atrial fibrillation; CT-ADP, closure time of adenosine diphosphate; MACCE, major adverse cardiac and cerebrovascular events.
Note: Values are *n* (%) or *n*/*N* (%).

 © 2023. The Author(s).

Supplementary Table S4 Predictors of major/life-threatening bleeding events at 1 year in patients without atrial fibrillation

	Univariate analysis		Multivariate analysis	
	HR (95% CI)	*p*-Value	HR (95% CI)	*p*-Value
Age, y	1.03 (0.99–1.08)	0.15		
Men	0.91 (0.52–1.59)	0.75		
Body mass index, kg/m^2	0.98 (0.92–1.04)	0.49		
Logistic EuroSCORE	1.01 (0.99–1.04)	0.24		
EuroSCORE 2	1.03 (0.96–1.08)	0.39		
STS score	1.05 (1.00–1.10)	0.07		
NYHA class III or IV	1.22 (0.70–2.18)	0.48		
Hypertension	1.90 (0.83–5.50)	0.13		
Dyslipidemia	1.03 (0.59–1.85)	0.92		
Diabetes mellitus	1.12 (0.62–1.95)	0.70		
CKD (eGFR <60 mL/min/1.73 m^2)	1.51 (0.86–2.70)	0.15		
Hemodialysis	1.9894e-9[a]	0.13		
Prior myocardial infarction	2.16 (1.06–4.07)	0.04	2.48 (1.19–4.75)	0.02
Prior PCI	1.54 (0.88–2.67)	0.13		
Prior CABG	1.08 (0.41–2.33)	0.86		
Peripheral artery disease	1.49 (0.80–2.64)	0.20		
Prior stroke	1.31 (0.60–2.56)	0.48		
COPD	0.68 (0.37–1.56)	0.39		
Medication at baseline				
DAPT	0.90 (0.48–1.59)	0.72		
Clopidogrel	0.90 (0.48–1.62)	0.74		
OAC	0.83 (0.14–2.67)	0.79		
VKA	1.9605e-9[a]	0.07		
DOAC	2.52 (0.41–8.11)	0.26		
Echocardiography data at baseline				
Mean aortic gradient, mmHg	1.02 (1.00–1.04)	0.02	1.02 (1.01–1.04)	0.009
LVEF, %	0.99 (0.97–1.01)	0.18		
Laboratory tests at baseline				
WBC, 10^3 /mm^3	1.02 (089–1.15)	0.78		
Hb, g/dL	0.81 (0.69–0.96)	0.02	0.83 (0.70–0.98)	0.03
Platelet count, 10^3 /mm^3	0.99 (0.99–1.00)	0.01	0.99 (0.99–1.00)	0.01
CRP, mg/dL	1.07 (0.95–1.16)	0.23		
Creatinine, μmol/L	1.00 (1.00–1.00)	0.95		
eGFR, mL/min/1.73 m^2	0.99 (0.98–1.00)	0.09		
CT-ADP, s[b]	1.01 (1.00–1.01)	0.005		
Procedural characteristics				
Femoral approach	0.48 (0.24–1.05)	0.06		
Prothesis type				
Balloon-expandable	0.76 (0.44–1.32)	0.33		
Self-expandable	1.32 (0.76–2.28)	0.33		
Prosthesis diameter, mm	0.97 (0.87–1.07)	0.52		
Sheath diameter, mm	1.08 (0.93–1.24)	0.30		

(*Continued*)

 © 2023. The Author(s).

Supplementary Table S4 (*Continued*)

	Univariate analysis		Multivariate analysis	
	**HR (95% CI)**	***p*-Value**	**HR (95% CI)**	***p*-Value**
Postprocedural characteristics				
24-h after TAVR				
CT-ADP, s	1.00 (1.00–1.01)	0.01		
CT-ADP >180 s	1.93 (1.10–3.35)	0.02	1.69 (0.96–2.96)	0.07
PRI-VASP, %	0.98 (0.97–1.00)	0.06		
Medication at discharge				
Aspirin	0.46 (0.14–2.84)	0.34		
Clopidogrel	0.56 (0.29–1.24)	0.14		
OAC	1.00 (0.24–2.72)	1.00		
VKA	1.41 (0.23–4.55)	0.65		
DOAC	0.63 (0.04–2.86)	0.62		

Abbreviations: CABG, coronary artery bypass grafting; CKD, chronic kidney disease; COPD, chronic obstructive pulmonary disease; CRP, C-reactive protein; CT-ADP, closure time of adenosine diphosphate; DAPT, dual antiplatelet therapy; DOAC, direct oral anticoagulant; eGFR, estimated glomerular filtration rate; Hb, hemoglobin; LVEF, left ventricular ejection fraction; NYHA, New York Heart Association; OAC, oral anticoagulant; PCI, percutaneous coronary intervention; PRI-VASP, platelet reactivity index by vasodilator-stimulated phosphoprotein; TAVR, transcatheter aortic valve replacement; VKA, vitamin K antagonist; WBC, white blood cell.

[a]Since no bleeding event was found in patients under hemodialysis, those under VKA, the hazard ratios were not available.

[b]Due to the collinearity between CT-ADP levels at baseline and after TAVR, CT-ADP level after TAVR was selected for the multivariate analysis.

 © 2023. The Author(s).

Supplementary Table S5 Predictors of major/life-threatening bleeding events at 1 year in patients with atrial fibrillation

Model 1				
	Univariate analysis		Multivariate analysis	
	HR (95% CI)	*p*-Value	HR (95% CI)	*p*-Value
Age, y	1.02 (0.99–1.06)	0.20		
Men	0.81 (0.53–1.23)	0.32		
Body mass index, kg/m^2	0.97 (0.93–1.02)	0.22		
Logistic EuroSCORE[a]	1.02 (1.00–1.03)	0.02	1.01 (1.00–1.02)	0.15
EuroSCORE 2	1.00 (0.97–1.03)	0.89		
STS score[a]	1.03 (1.00–1.06)	0.03		
NYHA class III or IV	1.04 (0.67–1.63)	0.87		
Hypertension	1.26 (0.70–2.50)	0.47		
Dyslipidemia	1.01 (0.66–1.56)	0.96		
Diabetes mellitus	1.03 (0.65–1.59)	0.89		
CKD (eGFR <60 mL/min/1.73 m^2)	0.95 (0.62–1.48)	0.81		
Hemodialysis	1.94 (0.48–5.17)	0.31		
Prior myocardial infarction	0.64 (0.29–1.25)	0.21		
Prior PCI	0.92 (0.58–1.43)	0.73		
Prior CABG	0.67 (0.28–1.34)	0.28		
Peripheral artery disease	0.71 (0.43–1.13)	0.15		
Prior stroke	1.67 (0.99–2.69)	0.06		
COPD	0.79 (0.41–1.40)	0.44		
Medication at baseline				
DAPT	1.16 (0.63–1.98)	0.62		
Clopidogrel	1.32 (075–2.18)	0.32		
OAC	0.87 (0.55–1.42)	0.57		
VKA	0.83 (0.55–1.26)	0.39		
DOAC	1.11 (0.68–1.75)	0.67		
Echocardiography data at baseline				
Mean aortic gradient, mmHg	1.00 (0.98–1.02)	0.95		
LVEF, %	1.00 (0.99–1.02)	0.68		
Laboratory tests at baseline				
WBC, 10^3 /mm^3	0.91 (0.82–1.01)	0.09		
Hb, g/dL	0.77 (0.69–0.87)	<0.001	0.79 (0.67–0.92)	0.002
Platelet count, 10^3 /mm^3	1.00 (1.00–1.00)	0.41		
CRP, mg/dL	1.12 (0.99–1.23)	0.06		
Creatinine, μmol/L	1.00 (1.00–1.00)	0.78		
eGFR, mL/min/1.73 m^2	1.00 (0.99–1.01)	0.68		
CT-ADP, s	1.00 (1.00–1.00)	0.60		
Procedural characteristics				
Femoral approach	1.79 (0.75–5.87)	0.21		
Prothesis type				
Balloon-expandable	1.19 (0.78–1.86)	0.43		
Self-expandable	0.84 (0.54–1.29)	0.43		
Prosthesis diameter, mm	0.97 (0.90–1.04)	0.43		

(*Continued*)

 © 2023. The Author(s).

Supplementary Table S5 (*Continued*)

Model 1				
	Univariate analysis		Multivariate analysis	
	HR (95% CI)	*p*-Value	HR (95% CI)	*p*-Value
Sheath diameter, mm	0.96 (0.85–1.08)	0.56		
Postprocedural characteristics				
24-h after TAVR				
CT-ADP, s	1.01 (1.00–1.01)	<0.001		
CT-ADP >180 s	3.98 (2.61–6.03)	<0.001	3.54 (2.09–5.98)	<0.001
PRI-VASP, %	0.99 (0.97–1.00)	0.06		
Medication at discharge				
Aspirin	0.77 (0.39–1.72)	0.49		
Clopidogrel	1.18 (0.72–1.86)	0.50		
OAC	0.70 (0.42–1.25)	0.22		
VKA	0.84 (0.55–1.27)	0.40		
DOAC	0.99 (0.63–1.53)	0.97		

Abbreviations: CABG, coronary artery bypass grafting; CKD, chronic kidney disease; COPD, chronic obstructive pulmonary disease; CRP, C-reactive protein; CT-ADP, closure time of adenosine diphosphate; DAPT, dual antiplatelet therapy; DOAC, direct oral anticoagulant; eGFR, estimated glomerular filtration rate; Hb, hemoglobin; LVEF, left ventricular ejection fraction; NYHA, New York Heart Association; OAC, oral anticoagulant; PCI, percutaneous coronary intervention; PRI-VASP, platelet reactivity index by vasodilator-stimulated phosphoprotein; TAVR, transcatheter aortic valve replacement; VKA, vitamin K antagonist; WBC, white blood cell.
[a]Due to the collinearity between logistic EuroSCORE and STS score, logistic EuroSCORE was selected for the multivariate analysis.

Model 2				
	Univariate analysis		Multivariate analysis	
	HR (95% CI)	*p*-Value	HR (95% CI)	*p*-Value
Age, y	1.02 (0.99–1.06)	0.20	1.02 (0.98–1.06)	0.38
Logistic EuroSCORE	1.02 (1.00–1.03)	0.02	1.01 (0.99–1.03)	0.21
Hypertension	1.26 (0.70–2.50)	0.47	0.60 (0.30–1.34)	0.20
CKD (eGFR <60 mL/min/1.73 m^2)	0.95 (0.62–1.48)	0.81	0.76 (0.45–1.33)	0.33
Prior stroke	1.67 (0.99–2.69)	0.06	1.29 (0.63–2.43)	0.47
OAC at baseline	0.87 (0.55–1.42)	0.57	1.09 (0.62–1.99)	0.76
Hb at baseline, g/dL	0.77 (0.69–0.87)	<0.001	0.76 (0.64–0.90)	<0.001
Postprocedural CT-ADP >180 s	3.98 (2.61–6.03)	<0.001	3.84 (2.22–6.66)	<0.001

Abbreviations: CKD, chronic kidney disease; CT-ADP, closure time of adenosine diphosphate; eGFR, estimated glomerular filtration rate; Hb, hemoglobin; OAC, oral anticoagulant; PCI, percutaneous coronary intervention.

 © 2023. The Author(s).

Supplementary Table S6 Predictors of major/life-threatening bleeding events at 1 year in the whole cohort

	Univariate analysis		Multivariate analysis	
	HR (95% CI)	*p*-Value	HR (95% CI)	*p*-Value
Age, y	1.03 (1.00–1.06)	0.03	1.02 (0.98–1.05)	0.34
Men	0.86 (0.62–1.20)	0.38		
Body mass index, kg/ m^2	0.98 (0.94–1.01)	0.22		
Logistic EuroSCORE[a]	1.02 (1.01–1.03)	0.005	1.01 (0.99–1.02)	0.37
EuroSCORE 2	1.01 (0.98–1.04)	0.42		
STS score[a]	1.04 (1.02–1.06)	0.003		
NYHA class III or IV	1.17 (0.83–1.66)	0.38		
Hypertension	1.48 (0.91–2.60)	0.12		
Dyslipidemia	1.01 (0.72–1.43)	0.95		
Diabetes mellitus	1.04 (0.73–1.47)	0.81		
CKD (eGFR <60 mL/min/1.73 m^2)	1.22 (0.87–1.74)	0.25		
Hemodialysis	1.08 (0.27–2.85)	0.89		
Prior atrial fibrillation[b]	1.55 (1.12–2.18)	0.009		
Prior myocardial infarction	1.13 (0.67–1.78)	0.63		
Prior PCI	1.12 (0.79–1.58)	0.51		
Prior CABG	0.82 (0.44–1.39)	0.47		
Peripheral artery disease	0.98 (0.67–1.41)	0.91		
Prior stroke	1.55 (1.01–2.30)	0.04	1.29 (0.73–2.14)	0.36
COPD	0.77 (0.45–1.25)	0.31		
Medication at baseline				
DAPT	0.87 (0.57–1.29)	0.50		
Clopidogrel	0.97 (0.64–1.42)	0.88		
OAC	1.37 (0.98–1.91)	0.06		
VKA	1.14 (0.78–1.62)	0.49		
DOAC	1.50 (0.95–2.26)	0.08		
Echocardiography data at baseline				
Mean aortic gradient, mmHg	1.01 (0.99–1.02)	0.28		
LVEF, %	1.00 (0.99–1.01)	0.51		
Laboratory tests at baseline				
WBC, 10^3 /mm^3	0.96 (0.88–1.04)	0.28		
Hb, g/dL	0.78 (0.71–0.86)	<0.001	0.81 (0.71–0.91)	<0.001
Platelet count, 10^3 /mm^3	1.00 (0.99–1.00)	0.02	1.00 (1.00–1.00)	0.65
CRP, mg/dL	1.08 (1.01–1.15)	0.04	1.05 (0.95–1.13)	0.28
Creatinine, μmol/L	1.00 (1.00–1.00)	0.64		
eGFR, mL/min/1.73 m^2	0.99 (0.98–1.00)	0.05		
CT-ADP, s	1.00 (1.00–1.00)	0.17		
Procedural characteristics				
Femoral approach	0.92 (0.54–1.70)	0.77		
Non-femoral approach	1.09 (0.59–1.86)	0.77		
Prothesis type				
Balloon-expandable	1.01 (0.72–1.43)	0.95		
Self-expandable	0.99 (0.70–1.38)	0.95		

(*Continued*)

 © 2023. The Author(s).

Supplementary Table S6 (*Continued*)

	Univariate analysis		Multivariate analysis	
	HR (95% CI)	*p*-Value	HR (95% CI)	*p*-Value
Prosthesis diameter, mm	0.97 (0.92–1.03)	0.38		
Sheath diameter, mm	1.01 (0.92–1.10)	0.85		
Postprocedural characteristics (24-h after TAVR)				
CT-ADP, s[b]	1.01 (1.00–1.01)	<0.001		
CT-ADP >180 s[b]	2.70 (1.93–3.76)	<0.001		
PRI-VASP, %	0.99 (0.98–1.00)	0.08		
4 groups according to AF and CT-ADP after TAVR				
Non-AF + CT-ADP ≤180 s	Reference	–	Reference	–
Non-AF + CT-ADP >180 s	1.94 (1.11–3.36)	0.02	1.78 (0.95–3.32)	0.07
AF + CT-ADP ≤180 s	1.48 (0.93–2.38)	0.095	1.24 (0.65–2.38)	0.51
AF + CT-ADP >180 s	5.85 (3.63–9.55)	<0.001	4.47 (2.41–8.36)	<0.001
Medication at discharge				
Aspirin	0.61 (0.34–1.23)	0.15		
Clopidogrel	0.68 (0.48–0.94)	0.02	1.11 (0.68–1.82)	0.67
OAC	1.38 (0.99–1.93)	0.05		
VKA	1.23 (0.86–1.75)	0.25		
DOAC	1.26 (0.83–1.85)	0.27		
Number of antithrombotic therapies				
0	Reference	–		
1	0.55 (0.15–3.50)	0.46		
2	0.29 (0.09–1.75)	0.15		
3	0.44 (0.11- 2.91)	0.44		

Abbreviations: AF, atrial fibrillation; CABG, coronary artery bypass grafting; CKD, chronic kidney disease; COPD, chronic obstructive pulmonary disease; CRP, C-reactive protein; CT-ADP, closure time of adenosine diphosphate; DAPT, dual antiplatelet therapy; DOAC, direct oral anticoagulant; eGFR, estimated glomerular filtration rate; Hb, hemoglobin; LVEF, left ventricular ejection fraction; NYHA, New York Heart Association; OAC, oral anticoagulant; PCI, percutaneous coronary intervention; PRI-VASP, platelet reactivity index by vasodilator-stimulated phosphoprotein; TAVR, transcatheter aortic valve replacement; VKA, vitamin K antagonist; WBC, white blood cell.

[a]Due to the collinearity between logistic EuroSCORE and STS score, logistic EuroSCORE was selected for the multivariate analysis.

[b]Prior AF and postprocedural CT-ADP were not included in the multivariate analysis because of the high collinearity with the four groups according to AF and CT-ADP after TAVR.

 © 2023. The Author(s).

Supplementary Table S7 Predictors of MACCE at 1 year in the whole cohort

	Univariate analysis		Multivariate analysis	
	HR (95% CI)	*p*-Value	HR (95% CI)	*p*-Value
Age, y	1.01 (0.99–1.03)	0.54		
Men	1.19 (0.91–1.55)	0.21		
Body mass index, kg/ m^2	0.99 (0.96–1.01)	0.38		
Logistic EuroSCORE[a]	1.01 (1.00–1.02)	0.049	1.01 (1.00–1.02)	0.23
EuroSCORE 2	1.01 (0.98–1.03)	0.50		
STS score[a]	1.03 (1.01–1.05)	0.007		
NYHA class III or IV	1.31 (1.00–1.75)	0.05		
Hypertension	0.82 (0.59–1.18)	0.28		
Dyslipidemia	1.04 (0.80–1.38)	0.76		
Diabetes mellitus	0.84 (0.62–1.11)	0.23		
CKD (eGFR <60 mL/min/1.73 m^2)	1.44 (1.09–1.92)	0.01	1.22 (0.84–1.80)	0.29
Hemodialysis	1.43 (0.57–2.95)	0.41		
Prior atrial fibrillation[b]	1.57 (1.20–2.05)	<0.001		
Prior myocardial infarction	1.23 (0.83–1.76)	0.29		
Prior PCI	1.32 (1.00–1.73)	0.049	1.14 (0.78–1.64)	0.50
Prior CABG	1.22 (0.80–1.78)	0.33		
Peripheral artery disease	1.07 (0.79–1.43)	0.65		
Prior stroke	1.28 (0.89–1.79)	0.18		
COPD	1.45 (1.02–2.00)	0.04	1.47 (0.93–2.25)	0.096
Medication at baseline				
DAPT	0.89 (0.64–1.22)	0.47		
Clopidogrel	1.16 (0.85–1.56)	0.35		
OAC	1.60 (1.22–2.08)	<0.001	1.75 (0.92–3.54)	0.09
VKA[c]	1.42 (1.07–1.87)	0.02		
DOAC	1.40 (0.97–1.97)	0.07		
Echocardiography data at baseline				
Mean aortic gradient, mmHg	0.99 (0.98–1.00)	0.02	1.01 (1.00–1.02)	0.49
LVEF, %	1.00 (0.99–1.01)	0.56		
Laboratory tests at baseline				
WBC, 10^3 /mm^3	1.01 (0.95–1.07)	0.82		
Hb, g/dL	0.94 (0.87–1.01)	0.11		
Platelet count, 10^3 /mm^3	1.00 (1.00–1.00)	0.75		
CRP, mg/dL	1.11 (1.05–1.17)	0.002	1.10 (1.02–1.18)	0.008
Creatinine, μmol/L[d]	1.00 (1.00–1.00)	0.02		
eGFR, mL/min/1.73 m^2[d]	0.99 (0.98–1.00)	0.003		
CT-ADP, s	1.00 (1.00–1.00)	0.47		
Procedural characteristics				
Femoral approach	0.56 (0.39–0.84)	0.006	0.53 (0.33–0.89)	0.02
Non-femoral approach	1.79 (1.19–2.58)	0.006	1.88 (1.12–2.99)	0.02
Prothesis type				
Balloon-expandable	1.09 (0.83–1.43)	0.55		
Self-expandable	0.92 (0.70–1.21)	0.55		

(*Continued*)

 © 2023. The Author(s).

Supplementary Table S7 (*Continued*)

	Univariate analysis		Multivariate analysis	
	HR (95% CI)	*p*-Value	HR (95% CI)	*p*-Value
Prosthesis diameter, mm	1.03 (0.99–1.08)	0.18		
Sheath diameter, mm	1.04 (0.96–1.11)	0.36		
Postprocedural characteristics (24h after TAVR)				
CT-ADP, s	1.00 (1.00–1.00)	0.53		
CT-ADP >180 s	1.15 (0.85–1.54)	0.36		
PRI-VASP, %	1.00 (0.99–1.00)	0.35		
4 groups according to AF and CT-ADP after TAVR				
Non-AF + CT-ADP ≤180 s	Reference	–	Reference	–
Non-AF + CT-ADP >180 s	1.10 (0.69–1.71)	0.68	1.37 (0.75–2.44)	0.29
AF + CT-ADP ≤180 s	1.45 (1.06–2.01)	0.02	0.81 (0.40–1.64)	0.55
AF + CT-ADP >180 s	1.95 (1.25–2.99)	0.004	1.33 (0.64–2.70)	0.44
Medication at discharge				
Aspirin	0.58 (0.35–1.02)	0.06		
Clopidogrel	0.63 (0.49–0.83)	<0.001	0.67 (0.49–1.55)	0.64
OAC	1.54 (1.18–2.02)	0.001	1.15 (0.47–2.77)	0.76
VKA[c]	1.47 (1.11–1.94)	0.008		
DOAC	1.17 (0.84–1.61)	0.34		
Number of antithrombotic therapies				
0	Reference	–		
1	0.92 (0.27–5.78)	0.91		
2	0.47 (0.15–2.86)	0.35		
3	0.71 (0.19–4.55)	0.67		

Abbreviations: AF, atrial fibrillation; CABG, coronary artery bypass grafting; CKD, chronic kidney disease; COPD, chronic obstructive pulmonary disease; CRP, C-reactive protein; CT-ADP, closure time of adenosine diphosphate; DAPT, dual antiplatelet therapy; DOAC, direct oral anticoagulant; eGFR, estimated glomerular filtration rate; Hb, hemoglobin; LVEF, left ventricular ejection fraction; MACCE, major adverse cardiac and cerebrovascular events; NYHA, New York Heart Association; OAC, oral anticoagulant; PCI, percutaneous coronary intervention; PRI-VASP, platelet reactivity index by vasodilator-stimulated phosphoprotein; TAVR, transcatheter aortic valve replacement; VKA, vitamin K antagonist; WBC, white blood cell.

[a]Due to the collinearity between logistic EuroSCORE and STS score, logistic EuroSCORE was selected for the multivariate analysis.

[b]Prior AF was not included in the multivariate analysis because of the high collinearity with the four groups according to AF and CT-ADP after TAVR.

[c]Due to the collinearity between OAC and VKA, OAC was selected for the multivariate analysis.

[d]Due to the collinearity among CKD, Cr, and eGFR, CKD was selected for the multivariate analysis.

 © 2023. The Author(s).

Supplementary Table S8 Type of late major/life-threatening bleeding complications at 1 year after transcatheter aortic valve replacement

Bleeding location	Number of event (%)
Gastrointestinal	21 (62)
Intracranial	3 (9)
Nose or mouth	3 (9)
Perioperative bleeding	2 (6)
Skin or muscle	2 (6)
Urogenital	2 (6)
Access site	1 (3)

Abbreviation: TAVR, transcatheter aortic valve replacement.

Supplementary Table S9 Clinical events following transcatheter aortic valve replacement according to the number of antithrombotic therapies at discharge

Numbers of antithrombotic therapies at discharge	None ($n=5$)	One ($n=49$)	Two ($n=787$)	Three ($n=36$)	*p*-Value
In-hospital events ($n=877$)					
Major vascular complication	1 (20)	4 (8)	65 (8)	7 (19)	0.10
Annulus rupture	0 (0)	0 (0)	0 (0)	0 (0)	–
Coronary obstruction	0 (0)	0 (0)	2 (0.3)	1 (3)	0.08
Conversion to open heart surgery	0 (0)	0 (0)	0 (0)	0 (0)	–
Pericardial effusion	0 (0)	1 (2)	8 (1)	0 (0)	0.82
Major/life-threatening bleeding	2 (40)	11 (22)	106 (13)	8 (22)	0.06
Major bleeding	1 (20)	6 (12)	75 (10)	7 (19)	0.21
Life-threatening bleeding	1 (20)	7 (14)	33 (4)	1 (3)	0.004
Minor bleeding	1 (20)	13 (27)	143 (18)	12 (33)	0.08
Transfusion $\geq$2 units	2 (40)	16 (33)	123 (16)	9 (25)	0.005
1-year outcome ($n=873$)					
All cause death	1/5 (20)	7/48 (15)	88/783 (11)	5/36 (14)	0.79
Cardiovascular death	1/5 (20)	4/48 (8)	45/781 (6)	1/36 (3)	0.40
Cerebrovascular event	1/5 (20)	7/48 (15)	46/783 (6)	1/36 (3)	0.04
Myocardial infarction	0/5 (0)	0/48 (0)	12/783 (2)	2/36 (6)	0.22
Heart failure hospitalization	0/5 (0)	7/48 (15)	88/783 (11)	5/36 (14)	0.72
MACCE	2/5 (40)	18/48 (38)	185/783 (24)	12/36 (33)	0.08
Major/life-threatening bleeding	2/5 (40)	13/48 (27)	116/783 (15)	8/36 (22)	0.04
2-year outcome ($n=664$)					
All cause death	2/5 (40)	10/34 (29)	151/593 (25)	8/31 (26)	0.85
Cardiovascular death	1/5 (20)	5/34 (15)	70/591 (12)	3/31 (10)	0.87
Cerebrovascular event	1/5 (20)	4/34 (12)	44/593 (7)	2/31 (6)	0.57
Myocardial infarction	0/5 (0)	1/34 (3)	13/593 (2)	1/31 (3)	0.95
Heart failure hospitalization	0/5 (0)	6/34 (18)	107/593 (18)	7/31 (23)	0.68
MACCE	3/5 (60)	18/34 (53)	233/593 (39)	15/31 (48)	0.25
Major/life-threatening bleeding	2/5 (40)	8/34 (24)	108/593 (18)	9/31 (29)	0.25

Abbreviation: MACCE, major adverse cardiac and cerebrovascular events.
Note: Values are *n* (%) or *n*/*N* (%).

 © 2023. The Author(s).
